# Environmental *Mycobacterium avium* subsp. *paratuberculosis* Hosted by Free-Living Amoebae

**DOI:** 10.3389/fcimb.2018.00028

**Published:** 2018-02-09

**Authors:** Ascel Samba-Louaka, Etienne Robino, Thierry Cochard, Maxime Branger, Vincent Delafont, Willy Aucher, Wilfrid Wambeke, John P. Bannantine, Franck Biet, Yann Héchard

**Affiliations:** ^1^Université de Poitiers, Laboratoire Ecologie et Biologie des Interactions, UMR Centre National de la Recherche Scientifique 7267, Equipe Microbiologie de l'Eau, Poitiers, France; ^2^Institut National de la Recherche Agronomique, Université de Tours, UMR1282, Infectiologie et Santé Publique, Nouzilly, France; ^3^National Animal Disease Center, Agricultural Research Service, United States Department of Agriculture, Ames, IA, United States

**Keywords:** paratuberculosis, amoebae, water, infection, *Mycobacterium*, *Rosculus*

## Abstract

*Mycobacterium avium* subsp. *paratuberculosis* is responsible for paratuberculosis in animals. This disease, leading to an inflammation of the gastrointestinal tract, has a high impact on animal health and an important economic burden. The environmental life cycle of *M. avium* subsp. *paratuberculosis* is poorly understood and several studies suggest that free-living amoebae (FLA) might be a potential environmental host. FLA are protozoa found in water and soil that are described as reservoirs of pathogenic and non-pathogenic bacteria in the environment. Indeed, bacteria able to survive within these amoebae would survive phagocytosis from immune cells. In this study, we assessed the *in vitro* interactions between several strains of *M. avium* subsp. *paratuberculosis* and *Acanthamoeba castellanii*. The results indicate that the bacteria were able to grow within the amoeba and that they can survive for several days within their host. To explore the presence of *M. avium* subsp. *paratuberculosis* in environmental amoebae, we sampled water from farms positive for paratuberculosis. A *M. avium* subsp. *paratuberculosis* strain was detected within an environmental amoeba identified as related to the poorly described *Rosculus* genus. The bacterial strain was genotyped, showing that it was similar to previous infectious strains isolated from cattle. In conclusion, we described that various *M. avium* subsp. *paratuberculosis* strains were able to grow within amoebae and that these bacteria could be found on farm within amoebae isolated from the cattle environment. It validates that infected amoebae might be a reservoir and vector for the transmission of *M. avium* subsp. *paratuberculosis*.

## Introduction

*Mycobacterium avium* subsp. *paratuberculosis* (Map), the etiologic agent of paratuberculosis also called Johne's disease, induces extensive inflammation in the gastrointestinal tract of ruminants. This inflammation prevents absorption of nutrients, which leads to a chronic and progressive weight loss despite eating habits. The hallmark of advance stage disease in cattle is copious diarrhea, appearance of ribs and other skeletal bones. Paratuberculosis is responsible for economic losses up to $1.5 billion annually to the dairy industries on the five continents (Ott et al., 1999; Sweeney et al., 2012). The management of this epidemic, is particularly difficult because efficient prophylactic tools are lacking. There is no treatment and the vaccine available is not very effective and not widely used because it compromises the diagnosis of bovine tuberculosis. Serological diagnostic tools lack specificity and are not adapted to early diagnosis of an infection, although this is beginning to change with the discovery of new antigens (Li et al., 2017). Another factor complicating the management of this epidemic involves the shedding of the bacteria in the environment where it is able to survive. Map is a genetically homogenous subspecies of *M. avium*, especially among bovine, human and wildlife isolates. However, two prominent lineages of Map have emerged following molecular strain typing and comparative genomic analysis—ovine (Map-S) and bovine (Map-C) strains (Biet et al., 2012). Initially, the Map-S and Map-C type strains were distinguished based on their molecular fingerprints using IS*1311* polymorphism, MLSSR typing and *hsp65* sequencing. However, beyond genetic typing, there are observed phenotypic differences including differences in their pigmentation, growth characteristics and lipopeptide structures in their cell walls (Bannantine et al., 2017). The environmental life cycle of Map is still poorly understood and at least one recent publication suggests that free-living amoebae (FLA) might be a potential environmental host (Salgado et al., 2015).

Free-living amoebae (FLA) are protozoa found in the same environmental niches as Map, including water and soil (Rodríguez-Zaragoza, 1994). FLA mainly feed on bacteria in the environment and digest them by phagocytosis. They thus play, along with other grazing protists, a key role in shaping the bacterial community composition in the environment (Jürgens and Matz, 2002). However, it has been repeatedly shown over the last decades that some bacteria may resist this digestion and persist or even grow within amoebae (Greub and Raoult, 2004). Importantly, these resistant bacteria are generally also more resistant to phagocytic immune cells, such as macrophages. This is well documented for *Legionella pneumophila* and for various *Mycobacterium* species in interaction with *Acanthamoeba castellanii*. The latter is the most common species in water and the best known FLA. As a consequence, FLA are considered as a training ground for pathogenic bacteria, including some *Mycobacterium* species (Molmeret et al., 2005; Salah et al., 2009). The fate of internalized mycobacteria can drastically vary according to the species, ranging from digestion (e.g., *M. bovis* BCG) and survival without replication (e.g., *M. tuberculosis*) to intra amoebal multiplication (e.g., *M. abscessus* or *M. chelonae*) (Drancourt, 2014). However, as described in this paper, most of the interactions were studied *in vitro* and only few studies have reported mycobacteria detected inside amoebae isolated from the environment (White et al., 2010; Amissah et al., 2014). Our group has previously described such interaction in drinking water (Delafont et al., 2014). Importantly, intra-amoebal growth of *Mycobacterium* would lead to improve its virulence (Cirillo et al., 1997).

The interaction between Map and amoebae has been poorly described. It was first reported in 2006 through a study showing *in vitro* that a strain of Map was ingested by *Acanthamoeba* and resisted digestion for at least 24 days (Whan et al., 2006). Four years later a short communication described the co-occurrence of Map and FLA in the soil (White et al., 2010). Amoebae and Map were found in the same soil samples and an amoeba strain was isolated harboring an intracellular acid-fast stained bacterium. Mura et al. (2006) described *in vitro* that Map persisted for up to four years in presence of *Acanthamoeba* (Mura et al., 2006). Recently, Map were found within amoebae isolated from soil after application of cattle slurry spiked with Map (Salgado et al., 2015). However, these studies used a very limited range of strains and no study has demonstrated clearly the presence of Map in environmental FLA.

Our study was aimed to assess the infectious potential of several Map strains including the two main genetic lineages of Map, C and S types toward *A. castellanii*. We also investigated whether Map-infected FLA might be found in the environment surrounding infected cattle.

## Materials and methods

### Cultivation of microorganisms

Bacterial strains used in this study are listed in Table 1. Mycobacterial strains were grown at 37°C in Sauton medium or Middlebrook 7H9 broth (Difco Laboratories, Detroit, MI) with 0.2% glycerol and albumin-dextrose-catalase enrichment medium (ADC, Becton Dickinson, Le Pont de claix, France). *M. avium* subsp. *paratuberculosis* cultures were supplemented with 2 mg l^−1^ mycobactin J (Allied Monitor). Bacteria were harvested at mid-log phase and kept frozen (−80°C) in aliquots until use. Recombinant *M. avium* subsp. *paratuberculosis*, strain K-10 expressing the Green fluorescent protein (GFP) - and luciferase described in Lefrançois et al. (2011) was grown in the Middlebrook 7H10 medium supplemented with ADC, 0.2 mg ml-1 mycobactin and 50 mg ml^−1^ of hygromycin. *Escherichia coli* strain K12 was grown in LB liquid medium (Lysogeny Broth: 10 g/L NaCl, 5 g/L yeast extract, 10 g/L tryptone), and incubated for 24 h at 37°C, under agitation (180 rpm).

**Table 1:** Bacterial and amoebal strains.

**Strains**	**Description, type, and genotype**	**References or source**
**BACTERIAL STRAINS**		
K10–ATCCBAA-968	Bovine isolate, C type, ^a^INMV 2	Li et al., 2005
205	Bovine isolate, C type, INMV 13	Biet et al., 2008
397	Ovine isolate, S type, INMV 70	Bannantine et al., 2012
453	Bovine isolate, C type, INMV 2	This study
6796	Ovine isolate, S type INMV 72	This study
7912	Bovine isolate, C type, INMV 9	Biet et al., 2008
GFPLux-Map K10	Green fluorescent protein (GFP)- and luciferase-expressing strains Map k10	Lefrançois et al., 2011
*E. coli* K12 MG1655		ATCC 47076
**AMOEBA**		
*A. castellanii* ATCC 30010		ATCC

a Profiles described by Thibault et al. (2007) and available on

http://mac-inmv.tours.inra.fr/

The free-living amoeba *A. castellanii* ATCC 30010 was grown in PYG liquid medium (ATCC 712; 20 g/L proteose peptone, 1 g/L yeast extract, 1 g/L sodium citrate, 0.1 M D-glucose, 0.4 mM CaCl_2_, 4 mM MgSO_4_, 2.5 mM Na_2_HPO_4_, 2.5 mM KH_2_PO_4_, 50 μM Fe(NH_4_)_2_(SO_4_)_2_, pH 6.5) in 25 or 75 cm^2^ flask. *A. castellanii* cultures were incubated at 30°C for 3–4 days, until a cell confluency of 80–90% was reached.

### Isolation of environmental amoebae

Environmental water samples were collected in March 2016 from two farms located in “Indre et Loire” and reported to be contaminated with Map since more than 5 years. Two 1-L samples were taken for each herd from the drinking troughs. The samples were filtered through a 5 μm nitrocellulose membrane (N3771-100EA, Sigma-Aldrich). The membranes were placed onto NNA plates (Non Nutrient Agar, 15 g/L) seeded with *live E. coli* strain K12. The plates were incubated at 30°C and examined daily under phase-contrast microscopy. The presence of amoebal cells is characterized by the formation of a migration front, representing the amoebal movement upon agar plates, seeking for food. Amoebae were recovered by scrapping the agar plate at the migration front for collection or DNA extraction.

### *A. castellanii* infection by different genotypes of map

*A. castellanii* cells from fully grown flasks were rinsed once using PAS buffer [Page's Amoeba Saline; 1 g/L sodium citrate, 0.4 mM CaCl_2_, 4 mM MgSO_4_, 2.5 mM Na_2_HPO_4_, 2.5 mM KH_2_PO_4_, 50 μM Fe(NH_4_)_2_(SO_4_)_2_, pH 6.5], and resuspended in a mixture of PAS/PYG (1:1) supplemented with 0.005% triton X-100, to avoid mycobacteria clumping in the medium. Trophozoites cells were harvested and detached by vigorously tapping the flask. The cell suspension was adjusted to a concentration of 1.25 × 10^5^ cells/mL, and 2 mL of the suspension distributed in each well of a 12 well plates. Mycobacteria cells were dispersed by 3 passages through a 18 Gauge needle, followed by a centrifugation step at 200 g for 5 min. The suspensions were subsequently quantified by optical density (OD) at 600 nm, using an equivalence of 6 × 10^8^ mycobacteria per mL for 1 OD unit. Mycobacteria suspensions were used to infect *A. castellanii* at an MOI (multiplicity of infection) of 10, i.e., 10 bacteria per amoeba cell. The infected cultures were then centrifuged at 500 g for 10 min to facilitate the infection, and incubated for 1 h at 30°C. The supernatant was then discarded and directly replaced with a fresh mixture of PAS/PYG (1:1), 0.005% triton X-100, supplemented with amikacin at 10 μM. Timepoints at 1, 24, 48, and 72 h post infection at 30°C were selected to estimate presence or growth of Mycobacteria by qPCR. To estimate the growth of *A. castellanii*, cells were harvested at different time points and counted using plastic counting slides FastRead 102 (Biosigma).

The localization of bacteria within amoeba was visualized using a recombinant rGFP-Map strain K-10 (Table 1) previously described (Lefrançois et al., 2011). Infected cultures were analyzed by fluorescence microscopy (BX41, Olympus).

### Total genomic DNA extraction

Cells from infection experiments as well as from environmental amoebae isolated as described above, were used for DNA extraction. Amoeba cells (4 × 10^4^ - 2 × 10^6^) and associated mycobacteria were harvested and resuspended in 500 μL of PAS buffer. Cells were lysed by bead-beating in tubes containing 500 mg of small diameter glass beads (100 μm) and 4 glass beads of 2 mm diameter (Sigma) using Fastprep apparatus for 30 s (speed 5 m/s). The suspension was then processed for DNA extraction using NucleoSpin Microbial DNA (Macherey-Nagel), following manufacturer recommendations for bacterial DNA extraction.

### Detection and quantification of mycobacteria by quantitative PCR (qPCR)

The presence of bacterial genomic DNA was assessed by qPCR performed in a final volume of 10 μL, including 2 μL of extracted DNA, 2 μL of 5x Master Mix LightCycler® FastStart DNA Master^plus^ SYBR Green I (Roche), a set of primer pairs (Table 2), at a concentration of 0.5 μM each, and water in sufficient quantity for 10 μL. Experiments were carried out using a LightCycler 1.5 thermocycler (Roche), consisting in an initial denaturation step at 95°C for 10 min, 45 cycles of denaturation at 95°C for 10 s, annealing for 10 s at a temperature according to the primer pairs used, and elongation at 72°C for 20 s. Fusion curves were collected for each experiment. Quantification of DNA was performed by calculating fold changes using the 2^−ΔCt^ method. ΔCt corresponds to the difference in cycle threshold (Ct) between a specific time point and the condition at 1 h.

**Table 2:** Oligonucleotide primers.

**Name**	**Sequence**	**Melting temp. (°C)**	**References**
atpE-F	5′-CGGYGCCGGTATCGGYGA-3′	58	Radomski et al., 2013
atpE-R	5′-CGAAGACGAACARSGCCAT-3′		
IS*900*-F	5′-GACGCGATGATCGAGGAG-3′	58	Pillai and Jayarao, 2002
IS*900*-R	5′-GGGCATGCTCAGGATGAT-3′		
F57-F	5′-TTGGACGATCCGAATATGT-3′	56	Tasara and Stephan, 2005
F57-R	5′-AGTGGGAGGCGTACCA-3′		

### Multiple-locus variable number tandem repeat analysis (MLVA) typing of environmental mycobacteria, using MIRU-VNTR markers

The method has been described previously (Thibault et al., 2007). The PCR mixture was composed as follows using the Go Taq Flexi DNA polymerase (Promega). Five microliters from DNA solution were added to a final volume of 25 μL containing 0.1 μL of Go Taq Flexi DNA polymerase (5 U/μL), 5 μl of betaine (Sigma), or 1 μl of dimethyl sulfoxide (Sigma); 0.2 mM (each) dATP, dCTP, dGTP, and dTTP (Promega); 5 μL of 5 × PCR buffer supplied by the manufacturer; 1 μM of primers see (Thibault et al., 2007); and 1.5 mM of MgCl_2_. The reactions were carried out using a TC-520 thermal cycler (Techne). PCR conditions were as follows: 1 cycle of 5 min at 94°C; 30 cycles of 30 s at 94°C, 30 s at 55, 58, 60, 64°C according to MIRU-VNTR searched, and 30 s at 72°C; 1 cycle of 7 min at 72°C. To detect differences in repeat numbers, the PCR products were analyzed by electrophoresis using 1.5% agarose gels (agarose electrophoresis grade; Invitrogen).

### Amoebal identification

The identification of the amoeba isolated from the water troughs was performed through sequencing of a 18S rRNA gene amplicon. From the total genomic DNA extract, a PCR was performed using the universal F566 and R1200 eukaryotic primers, targeting the 18S rRNA gene (Hadziavdic et al., 2014). The amplicon was sequenced and the sequence compared to the nucleotide nr database using BLASTn.

### Evaluation of phagosomal acidification

Mycobacteria suspension prepared as described previously were pre-labeled using pHrodo^TM^ Red succinimidyl ester (ThermoFischer Scientific) following manufacturer recommendations, but excluding the methanol washing step. Briefly, bacterial suspensions were incubated for 1 h in 0.1 M sodium bicarbonate buffer containing 20 μM pHrodo^TM^ Red succinimidyl ester, in the dark. Bacteria were then pelleted by centrifugation at 12,000 g for 5 min, and washed twice in PAS buffer supplemented with 0.005% triton X-100. *A. castellanii* monolayers were infected by labeled mycobacteria at a MOI of 10. After 4 h of incubation, trophozoites were detached, fixed with 2% paraformaldehyde for 10 min in the dark. Cells were pelleted by centrifugation at 800 g for 10 min, and resuspended in a 30 μL of SlowFade Diamonds antifade mountant (ThermoFischer Scientific) with Hoechst at a concentration of 400 ng/mL. Samples were examined using an epifluorescence microscope (BX41, Olympus).

### Statistics

Results were statistically analyzed using GraphPad Prism software. Multiple comparison was performed using Kruskal–Wallis analysis followed by a Dunn's post-test. Differences with *p* < 0.05 were considered statistically significant.

## Results

### Map strains grow within *A. castellanii*

**Figure 1:**
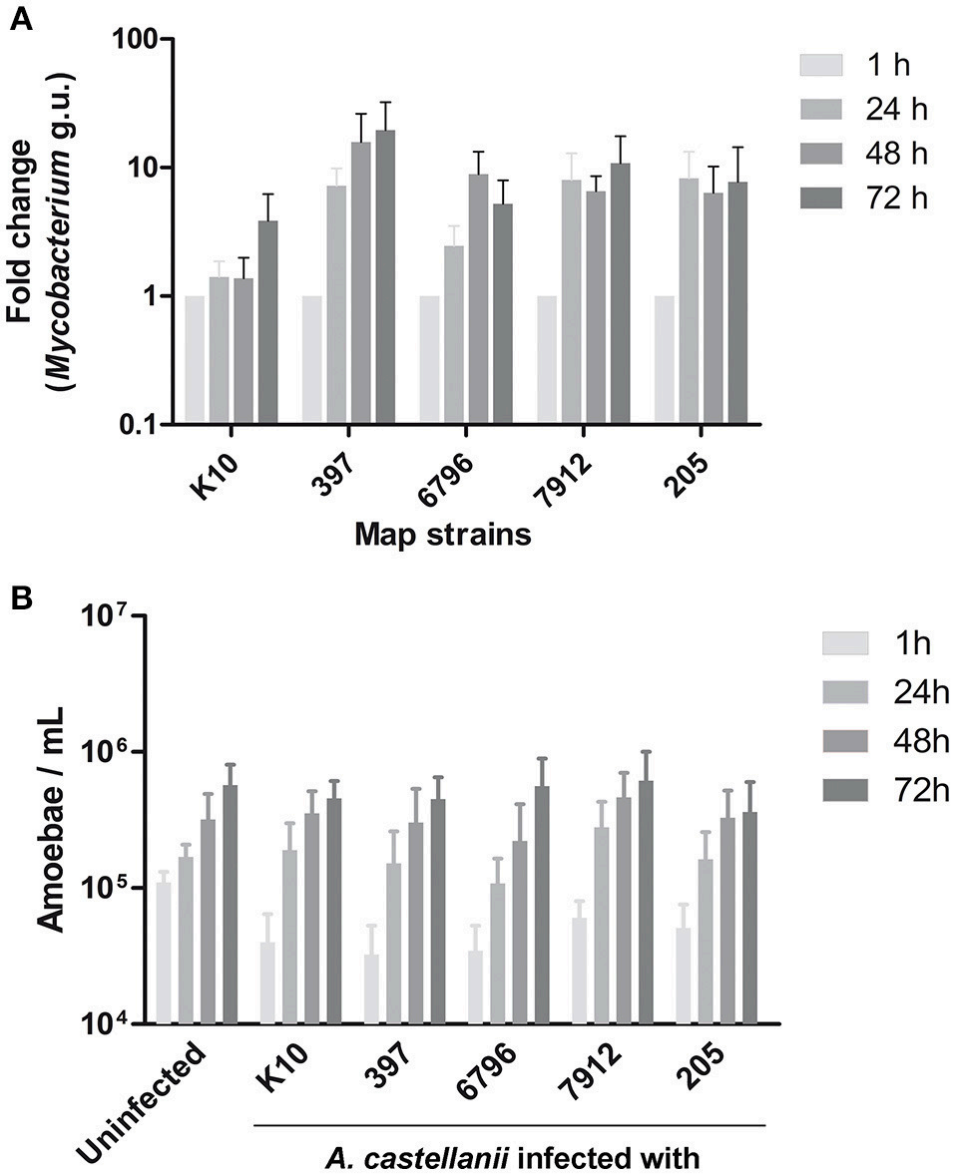
*A. castellanii* is permissive to both ovine (S) and bovine (C) Map. **(A)** *A. castellanii* were infected with ovine (397, 6796) and bovine (K10, 7912, 205) strains of Map at a MOI of 10. Presence of *Mycobacterium* spp. was quantified (*Mycobacterium* genome unit) through qPCR amplification of the *atpE* gene, 1, 24, 48, and 72 h post-infection. Results represents the mean (±SEM) of four independent experiments (*p* = 0.4441). Results are normalized on the condition 1 h. **(B)** Uninfected and infected *A. castellanii* were counted 1, 24, 48, and 72 h post-infection. Results represents the mean (±SEM) of four independent experiments (*p* = 0.8939).

In order to see whether Map strains would behave differently within amoeba, we investigated infection of *A. castellanii* by different genotype and genetic lineage of Map strains. *A. castellanii* were co-cultured with the reference strain (K10), two cattle strains (205, 7912), and two sheep strains (397, 6796). The fate of Map internalized by *A. castellanii* was determined by a qPCR based bacterial quantification assay, at 1, 24, 48, and 72 h post infection. We found that all Map persisted and even grew within *A. castellanii* (Figure 1A). There were no significant differences in growth rate between the strains (*p* = 0.4441) even though the K10 strain tended to be slightly slower. The fate of infected *A. castellanii* was also monitored throughout the infection, indicating that the host proliferation was not significantly modified (*p* = 0.8939) by the presence of Map in comparison with an uninfected *A. castellanii* culture (Figure 1B). Taken together, these results suggest that Map can successfully infect and grow within *A. castellanii* without any deleterious effect on its host.

### Map resides within phagosomal compartments

**Figure 2:**
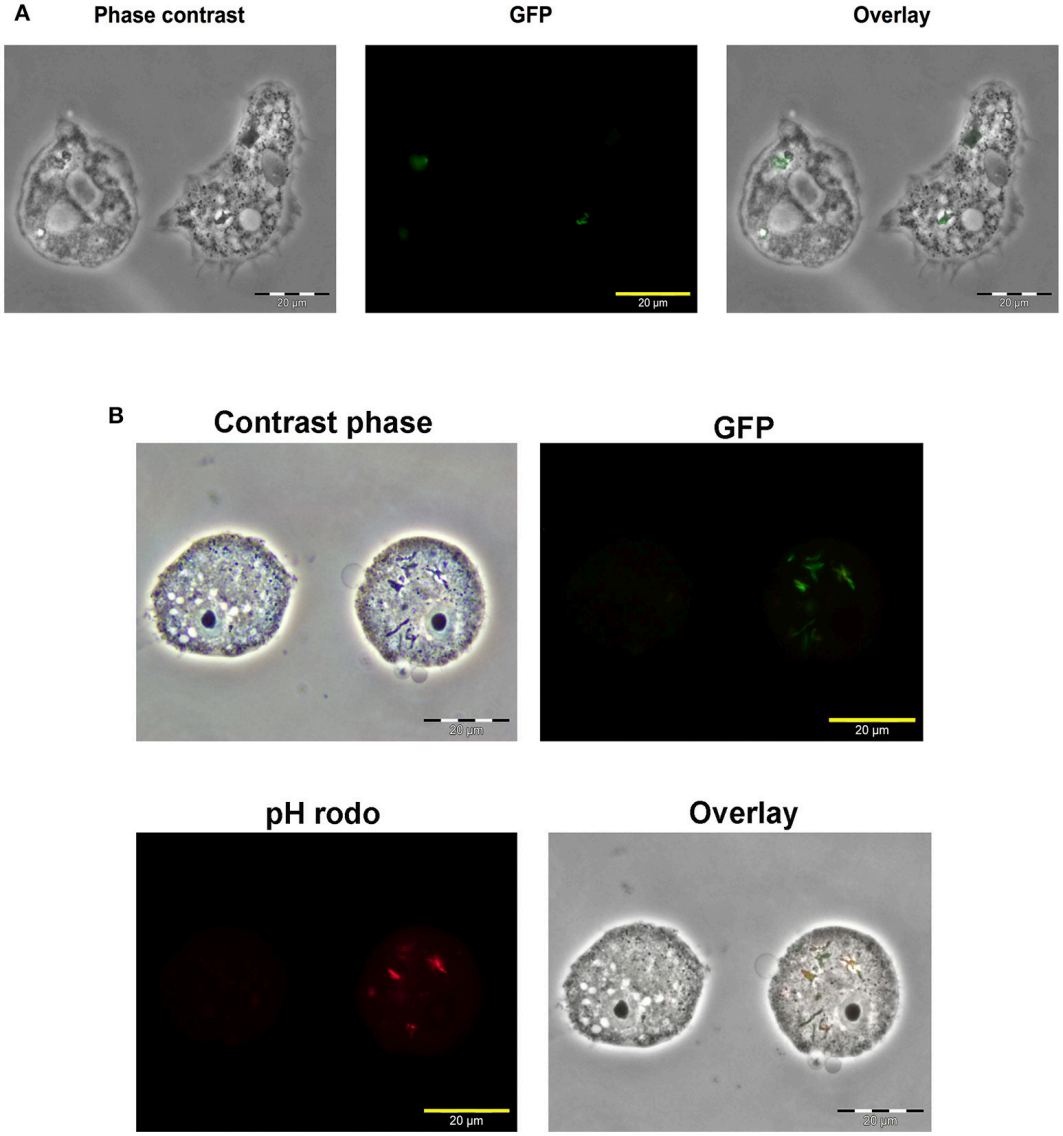
Map K10 persists within *A. castellanii* for up to 7 days. **(A)** *A. castellanii* were co-cultured with a K10-GFP map strain at a MOI of 10. Mycobacteria localization within amoebae trophozoites was assessed by epifluorescence microscopy 7 days after the initial infection. **(B)** *A. castellanii* were infected with K10-GFP labeled with a pHrodo dye at a MOI of 10. At 4 h post-infection, infected cells were analyzed through epifluorescence microscopy to reveal the Map K10 strain and acidification of bacteria through GFP and red signals.

To localize Map within *A. castellanii*, amoebae were infected with a Map K10 strain expressing a green fluorescent protein (GFP). Microscopy analysis of infected *A. castellanii* showed colocalization between the Map K10-GFP and *A. castellanii* 7 days after infection (Figure 2A). This figure also suggests that the bacteria were found inside *A. castellanii* vesicles. Later observations allowed to visualize *A. castellanii* cysts but no GFP signal from the K10-GFP strain was detected, suggesting that in our conditions Map K10 was not present within the cysts. We have also assessed the acidification of Map-containing intracellular compartments in the first hours of infection. Before infection, the Map K10-GFP strain was stained with the pHrodo dye, which fluoresces in a pH-dependent manner. The results show that a fraction of intracellular Map was labeled in red, indicating that they were found within acidified vacuoles, likely phagosomes, while other Map were not labeled (Figure 2B). These observations suggest that Map are phagocytised by *A. castellanii* and resides, at least transiently, within acidified vacuoles.

### Map is found within an environmental amoeba in infected farm

As no study had isolated environmental FLA infected by Map so far, we investigated the potential Map-amoeba association in water from the drinking troughs in herds naturally infected by Map. From the two farms enrolled in this study, only one sample was positive for the presence of cultivable amoebae. Total genomic DNA extracted from the amoebal culture was used to detect the presence of Map DNA using a Map-specific qPCR assay, targeting the IS*900* and F57 genes (Table 3). The successful amplification of the specific genomic target highlighted the presence of *Map* in association with the isolated amoebal culture. The Map strains K10, 397, and 7912 were used as positive control and *M. llatzerenze* as negative control for all PCR-based assays (Table 3). We have previously established a database containing isolated and genotyped Map strains from feces of infected cattle (Holbert et al., 2015). Therefore, we genotyped the Map-positive DNA from the amoeba culture, using MLVA genotyping and compare it to our database. The result of the MLVA genotyping based on the eight classical loci MIRU VNTR (Thibault et al., 2007) gave a profile INMV 2 identical to that of the 453 strain isolated from feces of cattle (Table 4).

**Table 3:** Map-specific qPCR targeting the atpE, IS*900* and F57 genes.

**Strains**	**PCR target**		
	**atpEF/atpER^a^**	**IS*900*F/IS*900*R^a^**	**F57F/F57R^a^**
Map K10	+	+	+
Map 397	+	+	+
Map 7912	+	+	+
*M. llatzerenze*	+	–	–
Total DNAs from isolated amoeba	+	+	+

a *AtpE primer set is specific of Mycobacterium spp. IS900 and F57 primer sets are specific of Map*.

**Table 4:** MLVA genotyping of Map strains.

	**Number of repeats by MLVA loci^a^**								**Name**
**DNA**	**MIRU 292**	**MIRU X3**	**VNTR 25**	**VNTR 47**	**VNTR 3**	**VNTR 7**	**VNTR 10**	**VNTR 32**	**INMV**
K10	3	2	3	3	2	2	2	8	2
397	7	1	3	3	1	1	1	8	70
205	2	2	3	3	2	2	2	8	13
6796	4	1	3	3	1	1	1	8	72
7912	2	1	3	3	2	2	2	8	9
453	3	2	3	3	2	2	2	8	2
Amoeba total DNA	3	2	3	3	2	2	2	8	2

a MLVA analysis and profile identification according to web database application http://mac-inmv.tours.inra.fr/

Furthermore, in order to identify the amoeba isolate, we tried to subculture the strain but no subculture was observed. Thus, we performed a PCR targeting a portion of 18S rRNA coding gene. Sequencing of purified amplicon (1265 nucleotides, GenBank MG859939), indicated that the most closely related sequence to the isolate was *Rosculus ithacus*, a cercozoan amoeba, although the sequence identity was moderate (78%). Therefore, this amoebal isolate is likely to represent a newly described genus among the sainouroid clade.

## Discussion

A growing number of studies underlines the important role of amoebae in the carrying of pathogenic and non-pathogenic mycobacteria, as well as in providing an environmental source of mycobacterial infection. On a microbiological level, the question of on farm disease transmission is particularly relevant because of the characteristics of Map biology. Indeed the disease transmission happens through ingestion of Map then during the disease progression, animals excrete bacilli in their environment, ranging from a few and discontinuous bacterial excretions in asymptomatic phases to very high levels and continuously shedding at clinical stage of the disease (Magombedze et al., 2017). This means that even in the silent phases of the disease the animals can excrete Map in the environment and in particular in the water points of the herds. In this context, it was interesting to investigate whether Map can be hosted and survive in amoeba and if water points of infected herds could be a source of contamination of Map mediated by FLA. Some other pathogenic mycobacteria were able to survive in soil and this environment might be a source of infection (Ghodbane et al., 2014).

In this study, the five selected strains (C- and S-type) not only survived but also showed a growth trend within *A. castellanii* for at least 72 h. In previous studies the growth of Map was not so high in the first days post-infection. This difference could be due to the *Acanthamoeba* strains and the infection protocols that were used in each study (Mura et al., 2006; Whan et al., 2006). For example, the co-cultures were centrifuged to increase efficiency and better synchronize the infection process. These differences might also explain the higher levels of bacteria per amoeba we observed. Moreover, we demonstrated that Map can persist for at least 7 days within *A. castellanii*. Within Map there is little genetic variability nevertheless the evolutionary pattern deciphered by Turenne et al. (2006) and Alexander et al. (2009) showed that the Map sub-species evolved from a common ancestor *M. avium* subsp. *hominissuis* into two different genetic lineages associated with a host preference. We speak of a lineage of S-type for sheep and C-type for cattle. In this study the strains were selected for their difference in terms of genotype and to include representative S and C-type. Altogether these results suggest that amoeba may serve as a significant phagocytic model to further investigate comparable or distinct phenotypic traits obtained by each lineage S and C-type of Map.

We also observed that the growth of *A. castellanii* was not adversely affected by infection with any of the Map strains. This indicates that Map had no deleterious effects on amoebal viability. These results suggest that interactions between these two types of microorganisms could very likely happen in the environment. This stability could indeed favor Map resilience in the environment, providing a shelter against harsh conditions, as well as a potential substrate source for mycobacterial multiplication (Caire-Brändli et al., 2014; Barisch et al., 2015; Delafont et al., 2017).

Microscopy studies indicate that Map was localized within phagosomes and that some bacteria were found in acidified environment while others were not. This suggests that Map could block, to a certain extent, the normal acidification process of *A. castellanii* phagosomes. Inhibition of phagosomal acidification has been observed with other *Mycobacterium* species both in amoebae and macrophages (Kuehnel et al., 2001; Rohde et al., 2007; Delafont et al., 2017). Although much of the cell biology has been done on mycobacteria-macrophage interactions, further investigations would be required to better understand the fate of Map after its internalization in amoeba.

Amoeba-Map association in the environment has been poorly documented. To our knowledge, only one short communication has reported that amoeba isolated from soil were positive for Map-specific PCR but neither the Map nor the amoebae strains were characterized (White et al., 2010). In our study, two water troughs were selected for sampling, as those were providing the water source to distinct herds previously declared as infected by Map. We were able to recover cultivable FLA from one of the water troughs, confirming their presence in this environment. From the cultivated amoeba, the presence of Map DNA was detected using two specific primer sets by qPCR. This finding represents a strong argument in favor of interactions between amoebae and Map in environmental conditions. MLVA genotyping confirmed the identification of the species Map. The MLVA profile of the DNA was identical to that of an isolated strain from feces of a Map naturally infected cattle in the same farm (Holbert et al., 2015). These results not only confirm the presence of FLA harboring Map in the environment of animals on farm, but the identical genotypes recovered may also suggest a link between residence in amoeba and ability to infect cattle. Further investigations could be done to isolate the Map strains and improve the discrimination of genotype by combination of genotyping methods to ascertain the strains isolated from amoebae and animals are strictly identical. These results indicate that the strains might persist and circulate in the herd. They further suggest that fresh drinking water sources on farm may be important for healthy cows and that the problem of Johne's disease may be difficult to solve using herd management practices alone.

In addition, the identification of the amoebal isolate indicated that its closest sequence corresponds to *R. ithacus*. The latter is a coprophilic amoeba belonging to the sainouroid clade, as part of the Cercozoan radiation. Interestingly, protozoa from this clade were also identified in cow and sheep dung in a previous study (Bass et al., 2016). It is therefore not surprising to have identified such an amoeba from a drinking through providing cattle. However, we were not able to subculture this amoebal strain for some reason even after several try. It suggests that the culture method that was used is not fully efficient to subculture any FLA.

In conclusion, our study showed that various Map strains were able to persist and grow within *A. castellanii*, without observable deleterious effects on the host. A screening of environment surrounding Map-infected herds allowed to recover a free-living amoeba, representative of the sainouroid clade, from a drinking trough. This environmental amoeba harbored a Map strain that was genotyped. This study is bringing additional pieces of evidence reinforcing the role of amoebae in the persistence, and potential transmission, of pathogenic mycobacteria, including Map. This work should stimulate further studies focused on the characterization of environmental interactions between amoebae and pathogenic mycobacteria.

## Author contributions

All the authors have substantial contributions to the conception or design of the work; or the acquisition, analysis, or interpretation of data for the work; drafting the work or revising it critically for important intellectual content and final approval of the version to be published.

### Conflict of interest statement

The authors declare that the research was conducted in the absence of any commercial or financial relationships that could be construed as a potential conflict of interest.
